# Great Facilitation of Thirty Years of Reforestation with Mixed Species to Ecosystem Nitrogen Accumulation in Dry-Hot Valley in the Jinsha River

**DOI:** 10.3390/ijerph191912660

**Published:** 2022-10-03

**Authors:** Zhilian Gong, Yong Li, Luqing Liu, Shuang Deng

**Affiliations:** 1 Department of Environmental Engineering, College of Food and Biological Engineering, Xihua University, Chengdu 610039, China; 2 Faculty of Geosciences and Environmental Engineering, Southwest Jiaotong University, Chengdu 610059, China

**Keywords:** ecosystem nitrogen, deep soil, afforestation, allocation

## Abstract

Nitrogen is a key factor influencing ecosystem structure and function in reforestation, but knowledge of ecosystem nitrogen accumulation through reforestation with mixed species is limited. Especially in the dry-hot valley of the Jinsha River, no studies cover total ecosystem nitrogen accumulation in mature plantations and its allocation for difficulty in collecting tree roots and deep soil from dry red soil. In this study, nitrogen accumulation of seven mixed plantations in the dry-hot valley in the Jinsha River was studied after thirty years of reforestation with an analogous sites method. The results were as follows: (1) Soil nitrogen stocks decreased with depth in the soil profile. Deep soil nitrogen storage (20–80 cm) was significantly correlated with stand age (R^2^ = 0.752, *p* = 0.000; *n* = 7), accounting for 56–63% of total soil nitrogen storage and 43–47% of soil nitrogen accumulation in the dry-hot valley. (2) Total biomass nitrogen stock of the 30-year-old plantation was 1.22 t ha^−1^, 61 times that of degraded wasteland and 7.6 times that of natural recovery shrub grassland, and it recovered to the reference level of natural forest following 30 years of reforestation. (3) Total ecosystem nitrogen stock in the 30-year-old plantation was 12.72 t ha^−1^, 1.4 times the reference wasteland and 1.19 times the natural recovery shrub grassland. The contribution of soil nitrogen to ecosystem nitrogen storage and accumulation was 90% and 67%, respectively. Litter nitrogen accounted for 1.6% ecosystem nitrogen storage. It indicated that reforestation with mixed plantation of *Leucaena leucocephala* and other species greatly facilitated more ecosystem nitrogen accumulation, especially soil nitrogen (including deep compartment). Secondary biomass nitrogen, especially litter, could not be overlooked. This study filled the gap of ecosystem nitrogen storage and distribution during reforestation in the dry-hot valley. Mixed plantation with legume species such as *L. leucocephala* and other species and an important role of secondary biomass, especially litter in nitrogen accumulation, provided a reference for the strategy formulation of reforestation and forest nitrogen management in the dry-hot valley and other semi-arid or arid regions.

## 1. Introduction

Nitrogen is an essential soil nutrient element, which can greatly affect plant growth and yield, thus affecting the structure and function of ecosystem in reforestation [1,2]. Inconsistent direction and amount of soil nitrogen stock change following reforestation have been reported for being influenced by biotic factors including species and microbe activity and abiotic factors including climate, soil texture and pH, etc. [3,4,5,6,7,8]. Deep soil has proven to play a great role in the soil nitrogen storage and nitrogen accumulation [9,10]. In Chinese forests, the average nitrogen storage in 30–100 cm depth accounted for approximately 59.75% of the total nitrogen storage in the 0–100 cm soil layer [11]. Hu et al. (2018) found that nitrogen accumulation at 0–50 cm depth was 1.68 times that at 0–20 cm depth following cropland abandonment [12]. Deep soil also provided an extremely important resource for evaluating soil inorganic nitrogen pools and their relative availability [10]. Therefore, it is essential to research soil nitrogen dynamics following reforestation, especially to research deep soil nitrogen.

Plant biomass nitrogen, as an important source of active nitrogen, was key to the nitrogen cycle following ecological restoration [13]. Leaf and root as active nitrogen sources had high turnover rates, with values of about 33–95% and 10–55%, respectively. Small fluctuations in the nitrogen pool of the active organs (leaf and root) would cause tremendous changes to the nitrogen exchange fluxes in the ecosystem [14,15]. Due to the difference of climate, soil and other factors, biomass nitrogen pool changes greatly in most ecological regions of China, ranging from 0.54 to 1.25 t ha^−1^, accounting for 3–11% in the ecosystem [11]. In the past, most studies focused on the nitrogen pool of tree biomass or nitrogen concentration in plant organs during reforestation, and they scarcely covered secondary biomass nitrogen (biomass nitrogen of shrub, grass, litter and coarse woody debris defined as dead trees and broken branches with a base diameter more than 1 cm) [2,16,17]. A few studies have pointed out that the secondary biomass nitrogen of understory shrubs, herbs and litter cannot be ignored. Litter nitrogen is related to the return of above ground nitrogen to the soil, plays an important role in the nitrogen cycle of the ecosystem, and is considered to be an important part affecting soil nitrogen [18,19].

It is necessary to study ecosystem nitrogen stocks and allocation (including nitrogen compartments of soil and biomass) to get on well with ecological restoration and nitrogen management. Ecosystem nitrogen storage and allocation varied for such factors as various climatic region, vegetation types and soil [11,13]. Previous studies analyzed the factors above using redundancy analysis and found that explanations of climate to vegetation nitrogen, soil nitrogen and ecosystem nitrogen were all the highest among the factors above, indicating that climate was the most primary influencing factor [11,13]. The total nitrogen storage in China’s forest ecosystems varied from 10.33 t ha^−1^ to 23.11 t ha^−1^ among different climate regions and from 12.87 to 18.32 t ha^−1^ among different forest types in China [11]. The ratio of vegetation nitrogen to soil nitrogen (0–100 cm) ranged from 0.03 to 0.16 in China’s forest ecosystems [11]. Li et al., 2021 also found that nitrogen storage in various forest ecosystems in the central Yunnan Plateau ranged from 4.47 ± 0.94 t ha^−1^ in *Pinus yunnanensis* to 8.91 ± 1.83 t ha^−1^ in *Pinus armandii* [20]. Soil, plants and litter contributed an average of 86.88%, 10.27% and 2.85% to forest nitrogen storage, respectively [20]. 

The dry-hot valley of the Jinsha River, characterized by high evapotranspiration rate and severe soil erosion, is recognized as one of the most fragile regions in southwestern China [21]. Great efforts have been made to reforest degraded wastelands to improve ecosystem structure and function, including nitrogen accumulation. In the dry-hot valley of the Jinsha River, previous studies mainly focused on the nitrogen concentration of plant organs or surface soil nitrogen [16,17]. However, information of biomass nitrogen storage and deep soil nitrogen accumulation in reforestation were sparse. No studies covered total ecosystem nitrogen accumulation in mature plantations including soil nitrogen, tree biomass nitrogen and secondary biomass nitrogen and its allocation for difficulty in collecting tree roots and deep soil from dry red soil in the dry-hot valley. In this study, 9–30 years old plantations were selected in the dry-hot valley of the Jinsha River with adjacent wastelands, a natural recovery shrub grassland and a natural forest as comparisons. The main objectives of this study were to: (1) explore soil nitrogen accumulation in 0–80 cm soil profile during 30 years of reforestation with mixed species on degraded wasteland in the dry-hot valley and the contribution of deep soil (below 20 cm depth) to soil nitrogen accumulation; (2) evaluate the total ecosystem nitrogen accumulation of a 30-year-old plantation and allocation among different compartments including soil, tree biomass and secondary biomass compared with natural restoring shrub grassland and remnant natural forests to get a better choice of nitrogen sequestration in the dry-hot valley and to provide nitrogen management suggestions for the ecological restoration of degraded wastelands in the dry-hot valley and other semi-arid or arid regions. We hypothesized that (1) reforestation with mixed species on a former wasteland in the dry-hot valley enhanced soil nitrogen accumulation and deep soil played an important part in nitrogen accumulation; (2) biomass nitrogen stock increased rapidly and secondary biomass nitrogen should be counted; (3) ecosystem nitrogen storage in a 30-year-old plantation was significantly higher than that of natural restoring shrub grassland and reference wasteland, and it recovered to the reference level of natural forest. 

## 2. Materials and Methods

### 2.1. Study Sites

Since the 1980s, reforestation has been implemented on degraded wastelands mostly with *L. leucocephala* on a large scale in the dry-hot valley in the Jinsha River. In this study, seven mixed plantations of different ages (9–30 years) established with *L. leucocephala* and other species were selected, six in Ningnan county of Sichuan province and one in Dongchuan municipality of Yunnan province, which were located in the dry-hot valley of the lower Jinsha River. The climate, vegetation and soil parameters such as pH, bulk density, texture and soil organic carbon had been reported in our former studies in the study sites [22,23]. We used analogous sites (spatial) in place of temporal chronosequence and took paired measurements of the adjacent reference wastelands, natural recovery shrub grassland and natural forest to account for differences in soil types and land-use history among properties. Disturbed by local people’s activities, adjacent wastelands were set as the baseline before reforestation. Additionally, only one remnant of natural forest and one natural recovery shrub grassland, which were protected by soil conservation projects, were found near the plantations in the dry-hot valley. In spite of a little limitation for lack enough reference natural recovery shrub grasslands and reference natural forests for each plantation, the stands were all located in dry-hot value, with similar temperature, precipitation and soil type. The adjacent wastelands, natural recovery shrub grassland and a natural forest were sampled as the reference sites to emulate soil properties prior to reforestation, under natural succession conditions and at an optimum state of plantation forests, respectively. In order to study ecosystem nitrogen accumulation, a 30-year-old plantation, as the oldest one among the plantations, was studied and compared with adjacent reference wasteland, reference natural recovery shrub grassland and reference natural forest in Ningnan county. Three 10 m × 10 m plots were selected in each mixed plantation forest, each reference wasteland and reference natural recovery shrub grassland for data collection. Three 20 m × 20 m plots were selected from the natural forest stand. Details of the study stands are presented in Table 1 and Table 2.

### 2.2. Sampling and Analysis

In all stands, soil profiles were excavated up to 80 cm deep or to bedrock. Soil sampling followed our previous study about ecosystem carbon in these stands [23]. After removing the litter, soil samples were taken from depths of 0–20, 20–40, 40–60, and 60–80 cm, respectively. Soil nitrogen concentration was detected by modified Kjeldahl method from Lu (2002) [24] and the standard set by Ministry of Ecological and Environmental Protection [HJ 717–2014]. Briefly, one gram soil sample was digested with a catalyst mixture (H_2_SO_4_-K_2_SO_4_-CuSO_4_-Se). Then, the sample was distilled with 20 mL of NaOH. The distilled NH_3_ was absorbed by 20 mL H_3_BO_3_ and then was measured by titration with 0.01 N hydrochloric acid. Soil bulk density was measured by a 100 m^3^ stainless steel bucket within the same depth range [24]. Soil nitrogen storage for each soil layer was calculated by multiplying mean nitrogen concentration by bulk density and soil depth, and total soil nitrogen storage in the soil profile was finally estimated by summing soil nitrogen storage of all soil layers. Following the method of previous studies [9,12], soil nitrogen stock changes were calculated from the baseline before reforestation. In this study, adjacent reference wasteland, heavily disturbed by anthropogenic activities, was set as the baseline before reforestation. The soil nitrogen accumulation rate in each mixed plantation was calculated as the difference in soil nitrogen storage between each plantation and the adjacent reference wasteland, divided by the recovery time.

Biomass nitrogen was investigated in the stands of a 30-year-old plantation, adjacent reference wasteland, reference natural recovery shrub grassland and reference natural forest. Biomass sampling followed our previous study about ecosystem carbon in these stands [23]. The biomass of tree layer was estimated by the mean tree technique. In brief, six sampled trees with approximate mean diameter at breast height were destructively sampled, including roots, stems, branches and foliage, in a 30-year-old plantation and reference natural forest. The biomass of shrub and grass was estimated by a destructive sampling within 3 subplots of 4 m^2^ and 1 m^2^, respectively. Coarse debris and litter were collected and sampled within subplots of 25 m^2^ and 1 m^2^, respectively, along the diagonal of each plot. Fresh weight of all biomass component and samples were obtained in field. Samples were placed in labeled airtight bags and transported to the laboratory for moisture determination. Nitrogen concentration of different biomass component was measured by the modified Kjeldahl method [24]. Biomass nitrogen storage of each component was calculated by multiplying nitrogen concentration by the biomass dry weight.

### 2.3. Statistical Analyses

Analyses were performed using SPSS 20.0 (SPSS Inc.: Chicago, IL, USA) for Windows. Significance levels were set at *p* = 0.05 in all statistical analyses. Before data analysis, normality and homogeneity of variance was test. One-way ANOVAs followed by least significant difference (LSD) tests was used to analyze the differences of soil nitrogen storage, biomass nitrogen storage and total nitrogen storage among different vegetation types. The relationships between soil nitrogen storage and stand age was test by Pearson correlation analysis. 

## 3. Results

### 3.1. Soil Nitrogen Accumulation in 0–80 cm Soil Profile

**Figure 1:**
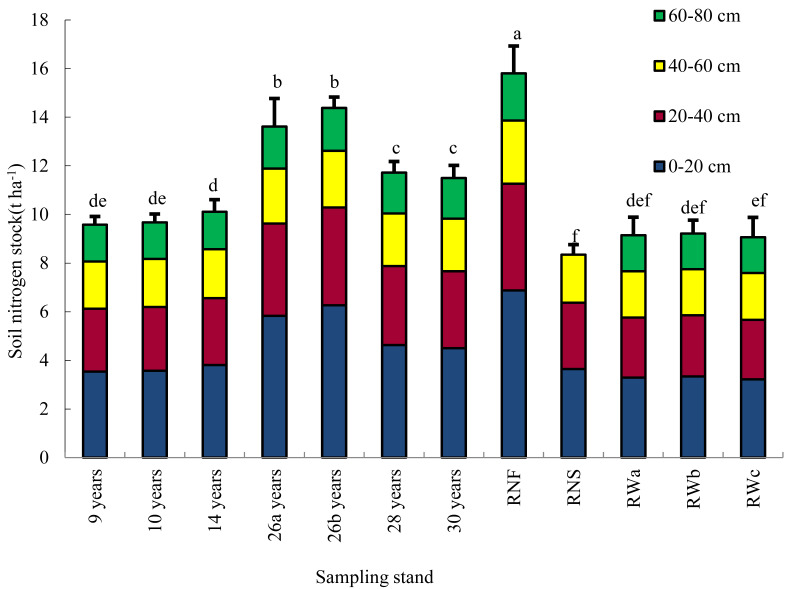
Soil nitrogen stocks at different depths in soil profile of plantations, reference wastelands, natural recovery shrub grassland (RNS) and reference natural forest (RNF) (t ha^−1^). Note: RWa, reference wasteland in Dongchuan municipality; RWb, RWc: reference wastelands in Ningnan county. 26a, mixed plantation with *L. leucocephala*, *Eucalyptus camaldulensis* and *Cajanus cajan*, aged 26. 26b, mixed plantation with *L. leucocephala* and *C. cajan*, aged 26. Data are means ± stand deviation, with the same letters among stands denote insignificant differences (*p* = 0.05).

Soil nitrogen storage at the depth of 0–20 cm, 20–40 cm, 40–60 cm and 60–80 cm in the plantations gradually decreased, with the average value of 4.59, 3.17, 2.12 and 1.63 t ha^−1^, respectively (Figure 1). Deep soil nitrogen storage (20–80 cm) of the plantations was 6.05–8.11 t ha^−1^, accounting for 56–63% of total soil nitrogen storage. Deep soil nitrogen storage was significantly correlated with stand age (R^2^ = 0.752, *p* = 0.000; *n* = 7).

Total soil nitrogen storage in plantations varied from 9.58 to 14.39 t ha^−1^ (Figure 1) and was correlated with plantation age significantly (R^2^ = 0.768; *p* = 0.044; *n* = 7), up to 1.6 times that of the reference wasteland, but still significantly lower than that of natural forest after 30 years of reforestation (15.80 t ha^−1^) (*p <* 0.05). Total soil nitrogen storage of mixed plantations in 0–60 cm soil profile was significantly higher than that of natural recovery shrub grassland after 26 years of reforestation (*p* = 0.000). Total soil nitrogen storage of reference wastelands and reference natural recovery shrub grassland was different insignificantly (*p* = 0.146). 

**Figure 2:**
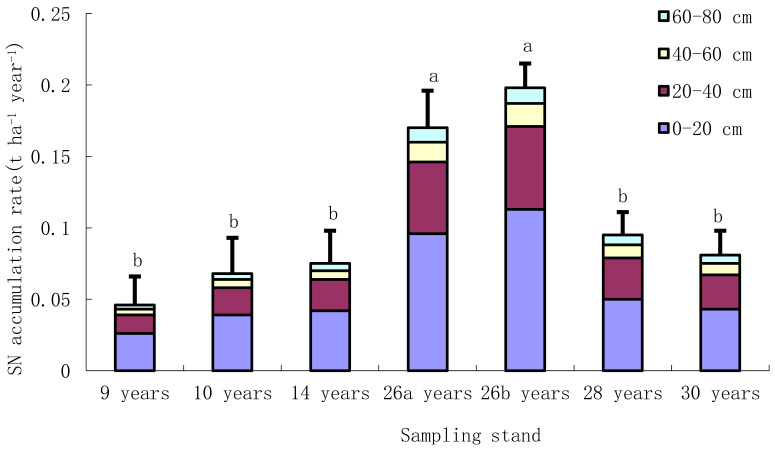
Soil nitrogen (SN) accumulation rate of plantations at different depths in soil profile. Note: 26a, mixed plantation with *L. leucocephala*, *E. camaldulensis* and *C. cajan*, aged 26; 26b, mixed plantation with *L. leucocephala* and *C. cajan*, aged 26. Data are means ± stand deviation, with the same letters among stands denote insignificant differences (*p* = 0.05).

In comparison to adjacent reference wasteland, soil nitrogen accumulation rate in 0–80 cm soil profile in mixed plantations ranged from 0.05 to 0.20 t ha^−1^ year^−1^, with an average value of 0.10 t ha^−1^ year^−1^ (Figure 2). Soil nitrogen accumulation rates at the depth of 20–80 cm ranged from 0.02 to 0.09 t ha^−1^ year^−1^, respectively, with the average value of 0.05 t ha^−1^ year^−1^, respectively (Figure 2). About 43–47% of soil nitrogen accumulation happened in deep soil layer in dry hot-valley. Soil nitrogen accumulation rate deep into rock bed (60 cm depth) in natural recovery shrub grassland was 0.022 t ha^−1^ year^−1^, with about 45% of Soil nitrogen accumulation in 20–60 cm soil layer. 

### 3.2. Biomass Nitrogen Storage

**Figure 3:**
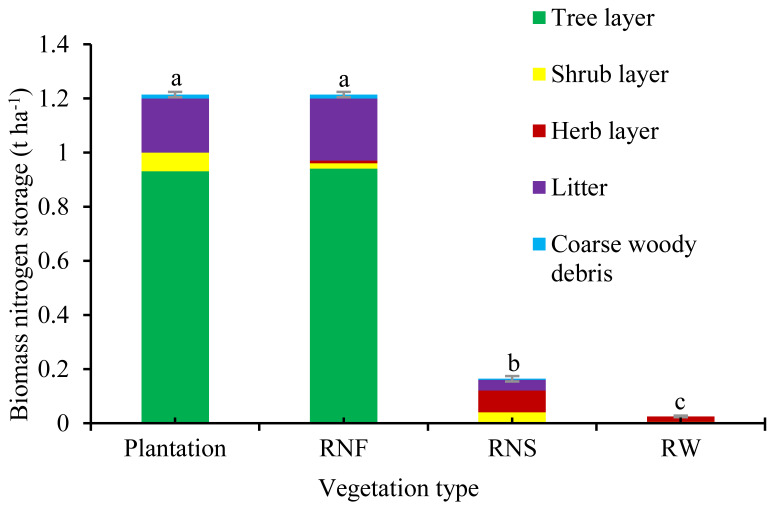
Biomass nitrogen stocks of different vegetation types. Note: Plantation: 30-year-old plantation; RNF: reference natural forest; RNS: reference natural recovery shrub grassland; RW: adjacent reference wasteland. Data are means ± stand deviation, with the same letters among stands denote insignificant differences (*p* = 0.05).

The total biomass nitrogen stock of a 30-year-old mixed plantation was 1.22 t N ha^−1^, 61 times that of degraded wasteland and 7.6 times that of natural recovery shrub grassland, but it did not significantly vary from that of natural forest (1.21 t ha^−1^) (*p* = 0.472) (Figure 3). The biomass nitrogen accumulation rate of a 30-year-old mixed plantation was 0.04 t ha^−1^ year^−1^, 10 times natural recovery shrub grassland.

Although the largest biomass nitrogen stock was in trees in both the 30-year-old plantation and natural forest, the secondary biomass nitrogen stocks still accounted for 24% and 22%, respectively. Nitrogen stock in litter compartment was the most important component of secondary biomass nitrogen storage. The rank of secondary biomass nitrogen stock in both forests was the same: litter compartment > shrub layer > coarse woody detritus compartment > herb layer. Biomass nitrogen of herb layer in a 30-year-old plantation was so little that it was not counted. The contribution of total root to biomass nitrogen in the 30-year-old plantation was 26%, higher than natural recovery shrub grassland (14%), but lower than reference natural forest (30%).

### 3.3. Total Ecosystem Nitrogen Storage

**Figure 4:**
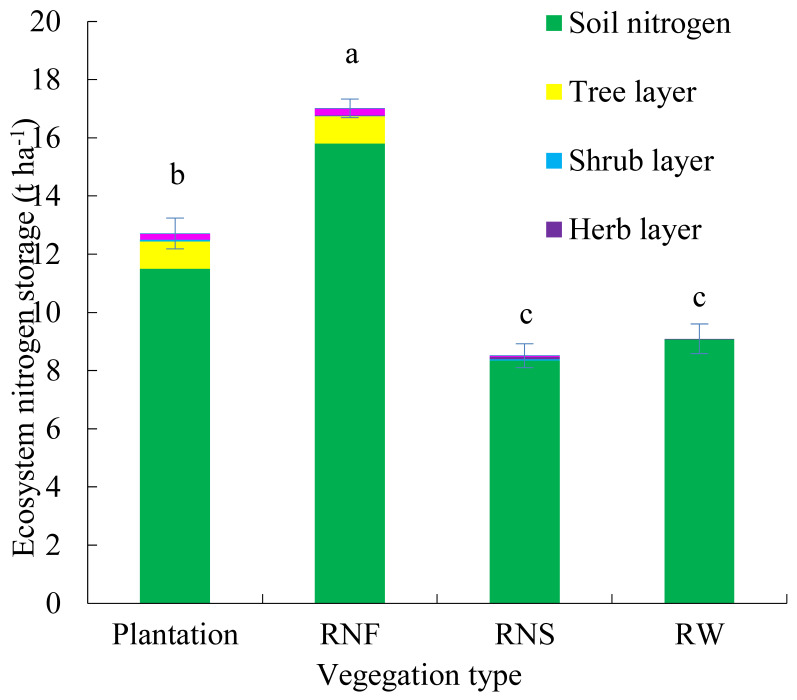
Ecosystem nitrogen storage of different vegetation types including nitrogen compartments of soil, tree biomass and secondary biomass. Note: Plantation: 30-year-old plantation; RNF: reference natural forest; RW: adjacent reference wasteland; RNS: reference natural recovery shrub grassland. Data are means ± stand deviation, with the same letters among stands denote insignificant differences (*p* = 0.05).

Total ecosystem nitrogen stock in a 30-year-old plantation was 12.72 t ha^−1^, 1.4 times that of reference wasteland, 1.19 times that of natural shrub grassland with the same soil depth, but 25% lower than that of natural forest (Figure 4). The largest ecosystem nitrogen compartment of the mixed plantation was in soil. Nitrogen stored in soil, tree layer and secondary biomass accounted for 90%, 8% and 2%, respectively. For natural forest, nitrogen stocks in soil, tree layer and secondary biomass accounted for 93%, 6% and 1%, respectively. For natural recovery shrub grassland and reference wasteland, much less nitrogen was stored in biomass, with the proportion of about 2% and 0%, respectively. Total ecosystem nitrogen accumulation rate of a 30-year-old plantation was 0.12 t ha^−1^ year^−1^. The contributions of biomass and soil to nitrogen accumulation were 33% and 67%, respectively.

## 4. Discussion

### 4.1. Nitrogen Accumulation in the Soil Profile after 30 Years of Reforestation

Soil nitrogen storage of the plantations gradually decreased with increasing soil depth of every 20 cm, which may be attributed to the decrease of nitrogen source from litter and plant roots along soil depth on one hand. The fine root biomass of dominant species *L. leucocephala* was proved to decrease with soil depth [25]. On the other hand, it might be due to variation of soil microbe abundance and composition along soil depth [26,27]. In the plantations of this study, carbon content decrease with soil depth (Table 2). Accordingly, it would be expected for heterotrophic microbes to decrease and for chemolithoautotrophs, such as the ammonia-oxidizing archaea to increase along soil depth [27]. The relative abundance of *Crenarchaeota*, playing crucial role in nitrification through ammonia oxidation, was found to increase to a depth of 60 cm [27]. Denitrification subtrate would increase, resulting in more nitrogen loss through denitrification in deep soil. 

As hypothesized, in the dry-hot valley, reforestation with mixed species on former wasteland enhanced soil nitrogen accumulation. Total soil nitrogen storage to 80 cm soil depth in mixed plantations varied from 9.58 to 14.39 t ha ^−1^, up to 1.6 times the reference wastelands and significantly higher than that of natural recovery shrub grassland after 26 years of reforestation (*p* < 0.01). Our result was comparable to the reports of some research in the areas under similar environmental constraints. Liu et al. (2018) found that soil nitrogen stock (0–30 cm) showed a significant increase after reforestation on wasteland in arid and semi-arid regions, up to 1.65 times the former wasteland. Reforestation on barren land with deciduous broadleaved species provided substantial opportunities for soil nitrogen sequestrations in arid and semi-arid regions [4]. On the Loess Plateau of China, plantations were found with higher soil nitrogen storage than grasslands following restoration from croplands [9]. In the loess hilly region of China, reforestation was proved to be a better choice than grassland for soil inorganic nitrogen restoration of degraded land [10].

The enhanced soil nitrogen accumulation of the mixed plantation in this study was possibly attributed to good restoration measures and stand characteristics. Firstly, different trees and shrubs with niche complementary effect were planted to make full use of resources and to gain great productivity. Meanwhile, legume species such as *L. leucocephala*, *Cajanus cajan* and *Tephrosia candida* were planted with non-legume species. Legume trees has been proved with extremely high nitrogen-fixing ability even on extremely contrasted soils. Percentage of nitrogen derived from atmosphere by a nitrogen-fixing species such as *Acacia spirorbis* could reach up to 80% [28]. Secondly, in this study, active biomass nitrogen such as litter and roots in the mixed plantations was higher than the natural recovery shrub grassland. Nitrogen stocks of litter and roots in the 30-year-old mixed plantation were 0.20 t ha^−1^ and 0.32 t ha^−1^, which is 5 times and 14 times that of natural recovery shrub grassland, respectively. The litter of dominant species *L. leucocephala* in this study, with thin, microphyllous and suitable C/N ratio, was easy to decomposition [5]. At the same time, root exudates have an obvious stimulating effect on nitrogen fixing bacteria in soil microorganisms, and nitrogen mineralization [29,30,31]. Lastly, lower soil pH in plantations compared with reference wastelands might be another factor facilitating soil nitrogen accumulation (Table 2). After reforestation, pH value and soil bulk density decreased with the increase of litter and root biomass, as a result of the forest soil biological process strengthening [32]. Low pH value limited the activity and quantity of nitrifying bacteria, inhibited nitrification and therefore reduced denitrification substrate and the release of gaseous nitrogen due to denitrification [3,33].

### 4.2. Nitrogen Accumulation in Deep Soil

This study showed that the deep soil of plantations in the dry-hot valley was an important soil nitrogen reservoir, and 56–63% of soil nitrogen is stored in 20–80 cm soil layer. This is similar to the results of previous studies. In China’s forest, the nitrogen storage in 30–100 cm depth accounted for approximately 59.75% of the total nitrogen storage in the 0–100 cm soil layer [11]. Li et al. (2019) found that soil at 40–200 cm accounted for 60–90% of the total inorganic nitrogen stock of the 0–200 cm soil profile along revegetation chronosequence in the loess hilly region of China [10].

The contribution of deep soil to total soil nitrogen accumulation increased with forest age, accounting for 47% of the total soil profile nitrogen accumulation after 30 years of reforestation. This may be related to the vertical distribution of fine roots of the dominant tree species *L. leucocephala.* Previous study found that the fine roots of *L. leucocephala* gradually developed towards deep expansion with the increase of forest age [31]. Fine root biomass of 3-, 5-, 9-, 14- and 20-year-old *L. leucocephala* in the 20–100 cm soil layer in the dry-hot valley accounted for 58%, 53%, 71%, 75% and 71% of the total fine root biomass in the 0–100 cm soil layer, respectively [25]. The nitrogen fixation of deep roots and root exudates promoted the deposition of deep soil nitrogen. The transport and retention of soil nitrogen in the vertical direction of soil layer was also an important source of deep soil nitrogen [34]. The clay content in the dry-hot valley was relatively low, and the infiltration rate of rainwater was fast, which promoted the migration of dissolved soil nitrogen to deep soil [3,7]. 

### 4.3. Biomass Nitrogen Accumulation

This study indicated that reforestation with mixed plantation of *L. leucocephala* and other species was a better choice of biomass nitrogen accumulation than grasslands. The total biomass nitrogen storage of the 30-year-old plantation was 1.22 t ha^−1^, 61 times that of degraded wasteland and 7.6 times that of naturally recovery shrub grassland and had recovered the reference level of natural forest after 30 years of reforestation. It was similar to the biomass carbon accumulation change in previous study [23]. Our result was higher than previous reports of younger plantations in the same region and mean biomass nitrogen storage in China’s forest [11,35]. Biomass nitrogen storages of 10-year-old *Azadirachta indica*, *Acacia auriculiformis* and mixed *A. indica*-*A. auriculiformis* were 0.07, 0.27 and 0.18 t ha^−1^ [35]. Biomass nitrogen stocks of China’s forests were averaged 0.86 t ha^−1^ [13]. The high biomass nitrogen stocks of mixed plantation in this study were probably due to its high forest age firstly. Besides, the dominant tree species *L. leucocephala* is a pioneer nitrogen fixation tree with fast growth and high generation rate. Secondly, the mixed plantation with niche complementary could make full use of limited resources, which was beneficial to biomass accumulation. Moreover, the secondary biomass nitrogen (including shrub layer, herb layer, litter and coarse woody debris) was taken into account in this study and it accounted for 27% of the plantation. In addition, plants adapted to the external environment and promoted their own growth by regulating the nitrogen content of each organ [2]. In the dry-hot valleys, *L. leucocephala* and other plants had high reabsorption rates of nitrogen, phosphorus and other restricted nutrients in infertile environments. Before littering, the leaves transferred nitrogen to the fresh leaves or roots to synthesize other substances, promoting plant to grow and adapt to the barren environment [16,35].

### 4.4. Nitrogen Accumulation in 30-Year-Old Plantation Ecosystem

Ecosystem nitrogen storage in the 30-year-old plantation was 12.72 t ha^−1^, 1.19 times higher than that of natural recovery shrub grassland, which is higher than the forest nitrogen storage in tropic humid region but lower than temperate arid region in China [11]. Previous studies found that forest nitrogen stocks were averaged 11.82 t ha^−1^ in tropic humid region and 15.46 t ha^−1^ in temperate arid regions in China [11]. Our result was also higher than the report in the central Yunnan Plateau. Nitrogen stocks in various forest ecosystems in the central Yunnan Plateau ranged from 4.47 ± 0.94 t ha^−1^ in *Pinus yunnanensis* to 8.91 ± 1.83 t ha^−1^ in *Pinus armandii* [20]. However, the ecosystem nitrogen stock of the 30-year-old plantation was still lower than that of reference natural forest, which was similar to ecosystem carbon changes of the plantation. It indicated that it would take longer time for ecosystem nitrogen of the mixed plantation to recover to the reference level of reference natural forest in the dry-hot valley.

Nitrogen stored in soil, tree layer and secondary biomass in the mixed plantation accounted for 90%, 8% and 2%, respectively, indicating that secondary biomass nitrogen, especially litter, was a component to be reckoned with in the plantation ecosystem nitrogen in the dry-hot valley. Litter nitrogen accounted for 1.6% ecosystem nitrogen in this study. In the dry-hot valley, most of the plants survived the dry season by litter. Our result was similar to the report in the central Yunnan Plateau. Li et al., 2021 found that litter contribution to ecosystem nitrogen storage varied from 1.1% to 4.5% in various forest ecosystems in the central Yunnan Plateau [20]. Litter, linking to the return of above ground nitrogen to the soil, played an important role in the nitrogen cycle of the ecosystem [18,19]. This study proved that secondary biomass nitrogen, especially litter should not be undervalued. We should enhance the management of secondary biomass nitrogen in the dry-hot valley.

## 5. Conclusions

The result of this study showed that soil nitrogen stocks in mixed plantations tended to decrease with soil depth increase in the dry-hot valley for the variation of microbial population and nitrogen source from litter and roots. However, 56–63% of total soil nitrogen storage and 43–47% of soil nitrogen accumulation happened at deep soil (20–80 cm). Total biomass nitrogen stock of the 30-year-old plantation was 61 times that of degraded wasteland and 7.6 times that of natural recovery shrub grassland. The enhanced nitrogen accumulation of mixed plantation may be due to niche complementary, good stand structure, and the characteristics of dominant species *L. leucocephala.* In the 30-year-old plantation, the contribution of soil, tree layer and secondary biomass to ecosystem nitrogen accounted for 90%, 8% and 2%, respectively. It indicated that secondary biomass nitrogen, especially litter, should be given importance in the dry-hot valley. This study indicated that compared with natural recovery shrub grassland, reforestation with mixed plantation of legume species such as *L. leucocephala* and other species facilitated more nitrogen accumulation whether in soil component or biomass component. The result of this study provided a guide for ecological restoration and forest nitrogen management in the dry-hot valley and other semi-arid or arid regions.

## Figures and Tables

**Table 1:** Description of the sampling stands.

Sites	Stand Description	Slope Aspect (°)	Slope (°)	Altitude (m)
Dongchuan municipality, Yunnan (N26°25′12′′; E103°04′43′′) (MAT: 22 °C; MAP: 700 mm)	9 years old plantation established with *Leucaena leucocephala*, *Acacia confusa*, *Eucalyptus camaldulensis* and *Dodonaea viscosa* (9 years)	NE80	20	895
	Reference wasteland in Tuobuka (RWa) (being wasteland for over 30 years)	NE80	20	910
Ningnan county, Sichuan (N27°04′15′′; 102.43′42′′) (MAT: 20–22 °C; MAP: 700–800 mm)	10 years old plantation established with *L. leucocephala* and *D. viscosa* (10 years)	NE63	18	860
	14 years old plantation established with *L. leucocephala* and *D. viscosa* (14 years)	NE66	19	800
	26 years old plantation established with *L. leucocephala*, *E. camaldulensis* and *Cajanus cajan* (26a years)	NE75	20	1273
	26 years old plantation established with *L. leucocephala* and *C. cajan* (26b years)	NE76	19	1273
	28 years old plantation established with *L. leucocephala* and *Tephrosia candida* (28 years)	NE60	17	840
	30 years old plantation established with 18 woody species, including *A. confusa*, *Bombaxceiba*, *E. camaldulensis*, *L. leucocephala*, *Tamarindus indica*, *Trema tomentosa*, *C. cajan* and *D. viscosa*(30 years)	NE70	18	805
	Reference wasteland near the two 26-year-old plantations (RWb) (being wasteland for over 46 years)	NE77	22	1260
	Reference wasteland near 10-,14-, 28-, 30-year-old plantations (RWc) (being wasteland for over 55 years)	NE35	21	821
	Reference natural recovery shrub grassland (RNS) (about 35 years old)	NE63	21	840
	Reference natural forest (RNF) (about 200 years old)	NE45	27	1230

**Table 2:** Soil parameters including pH and soil organic stocks at each depth along the soil profile.

Sites	Stand		pH			Carbon Stock (t ha^−1^)			
		0–20 cm	20–40 cm	40–60 cm	60–80 cm	0–20 cm	20–40 cm	40–60 cm	60–80 cm
Dongchuan municipality, Yunnan	9 years	7.6	7.5	7.9	8.0	32.55	21.64	14.31	10.76
	RWa	7.9	7.8	8.0	8.0	28.37	19.88	14.00	10.64
Ningnan county, Sichuan	10 years	7.5	7.6	7.7	8.0	33.51	22.70	16.16	12.00
	14 years	8.0	7.9	8.0	8.0	36.95	24.41	16.68	12.25
	26a years	5.9	5.7	5.6	5.8	63.39	37.25	20.23	14.65
	26b years	6.8	6.7	7.0	7.0	64.89	38.04	20.11	14.57
	28 years	7.6	7.4	7.3	7.3	46.45	29.48	19.08	13.24
	30 years	7.9	7.7	7.7	7.6	46.43	29.00	19.02	13.27
	RWb	7.5	7.4	7.7	7.7	31.43	22.16	15.63	11.74
	RWc	8.2	8.1	8.1	8.2	28.80	20.66	15.49	11.45
	RNS	8.0	7.8	7.7	/	32.62	23.66	16.35	/
	RNF	5.6	5.8	5.5	5.6	80.31	52.16	29.64	21.23
